# Characterizing Age-related Changes in Intact Mitochondrial Proteoforms in Murine Hearts using Quantitative Top-Down Proteomics

**DOI:** 10.21203/rs.3.rs-3868218/v1

**Published:** 2024-01-18

**Authors:** Andrea Ramirez-Sagredo, Anju Sunny, Kellye Cupp-Sutton, Trishika Chowdhury, Zhitao Zhao, si wu, Ying Ann Chiao

**Affiliations:** Oklahoma Medical Research Foundation; University of Alabama; University of Alabama; University of Alabama; University of Oklahoma; University of Alabama; Oklahoma Medical Research Foundation

**Keywords:** Top-down proteomics, label-free quantitation, post-translational modifications, mitochondria, cardiac aging

## Abstract

Cardiovascular diseases (CVDs) are the leading cause of death worldwide, and the prevalence of CVDs increases markedly with age. Due to the high energetic demand, the heart is highly sensitive to mitochondrial dysfunction. The complexity of the cardiac mitochondrial proteome hinders the development of effective strategies that target mitochondrial dysfunction in CVDs. Mammalian mitochondria are composed of over 1000 proteins, most of which can undergo post-translational protein modifications (PTMs). Top-down proteomics is a powerful technique for characterizing and quantifying all protein sequence variations and PTMs. However, there are still knowledge gaps in the study of age-related mitochondrial proteoform changes using this technique. In this study, we used top-down proteomics to identify intact mitochondrial proteoforms in young and old hearts and determined changes in protein abundance and PTMs in cardiac aging.

**METHODS:**

Intact mitochondria were isolated from the hearts of young (4-month-old) and old (24–25-month-old) mice. The mitochondria were lysed, and mitochondrial lysates were subjected to denaturation, reduction, and alkylation. For quantitative top-down analysis, there were 12 runs in total arising from 3 biological replicates in two conditions, with technical duplicates for each sample. The collected top-down datasets were deconvoluted and quantified, and then the proteoforms were identified.

**RESULTS:**

From a total of 12 LC-MS/MS runs, we identified 134 unique mitochondrial proteins in the different sub-mitochondrial compartments (OMM, IMS, IMM, matrix). 823 unique proteoforms in different mass ranges were identified. Compared to cardiac mitochondria of young mice, 7 proteoforms exhibited increased abundance and 13 proteoforms exhibited decreased abundance in cardiac mitochondria of old mice. Our analysis also detected PTMs of mitochondrial proteoforms, including *N*-terminal acetylation, lysine succinylation, lysine acetylation, oxidation, and phosphorylation.

**CONCLUSION:**

By combining mitochondrial protein enrichment using mitochondrial fractionation with quantitative top-down analysis using ultrahigh-pressure liquid chromatography (UPLC)-MS and label-free quantitation, we successfully identified and quantified intact proteoforms in the complex mitochondrial proteome. Using this approach, we detected age-related changes in abundance and PTMs of mitochondrial proteoforms in the heart.

## BACKGROUND

Aging is a physiological stage accompanied by the functional decline of multiple organs, including the heart. Aging also significantly increases the prevalence of cardiovascular diseases (CVDs) [1]. Heart function, especially diastolic function, declines progressively with age, and this baseline functional decline is accompanied by increased risks of pathological myocardial remodeling, cardiac hypertrophy, arrhythmia, microcirculatory dysfunction, and heart failure (HF) [2]. Due to the high energetic demand of the heart, heart function is tightly regulated by energy metabolism. Compromised mitochondrial structure, bioenergetics, and signaling have been observed in the aging heart [3]. Multiple mechanisms, including increased oxidative stress, mutations in mitochondrial DNA, and dysregulation of proteostasis have been shown to contribute to age-related mitochondrial dysfunction in the heart [4].

The mammalian mitochondrial proteome consists of more than 1000 different proteins [5]. Mitochondrial proteins can undergo post-translational modifications (PTMs) such as phosphorylation, acetylation, and oxidation. These PTMs add an additional layer of complexity to the mitochondrial proteome and modulate the properties and functions of mitochondrial proteins. PTMs of mitochondrial proteins can regulate intra-cellular signaling, mitochondrial energy generation, apoptosis, autophagy, and response to injury [6, 7]. Protein phosphorylation is one of the most studied PTMs and deregulated phosphorylation has been implicated in aging and diseases. For example, phosphorylation of cyclophilin D, a regulator of the mitochondrial permeability transition pore (mPTP), has been shown to sensitize mPTP opening and cell death after myocardial ischemia-reperfusion [8]. Nicotinamide adenine dinucleotide (NAD^+^) serves as a substrate for protein deacetylation by sirtuins (SIRTs). Cellular NAD^+^ levels decline with age [9, 10] and this decline plays a critical role in aging and age-related diseases [10–13]. Compared to young hearts, older hearts have decreased NAD^+^ levels and increased levels of total protein acetylation [14]. SIRT3 is the main protein deacetylase within the mitochondria and plays a key regulatory role in mitochondrial metabolism and signaling via protein deacetylation [7, 15]. Besides acetylation, SIRT5 has been shown to catalyze other NAD^+^-dependent lysine acyl modifications (i.e., malonylation, succinylation, and glutarylation) in the mitochondria [16]. The changes and functional roles of these PTMs in cardiac aging remain to be established.

Increased mitochondrial oxidative stress is a hallmark of cardiac aging and excess reactive oxygen species (ROS) promotes oxidative modifications on mitochondrial proteins [3]. Redox-related protein modifications like S-oxidation (sulfenylation and sulfinylation), S-nitrosylation, and S-glutathionylation have been shown to contribute to mitochondrial dysfunction in cardiomyocytes during cardiac aging [1, 17, 18]. However, further investigations are needed to expand our understanding of the roles of oxidative PTMs of specific mitochondrial proteins in cardiac aging.

Bottom-up proteomics is a powerful tool used to characterize and quantify differential protein expression as a result of disease, pharmaceutical treatment, environmental changes, phenotypic differences, etc. Bottom-up proteomics methods have even been applied to observe changes in PTMs primarily by implementing PTM-specific enrichment techniques or protein purification [1, 19]. However, since protein digestion is required for bottom-up methods, information relevant to the intact protein and proteoforms may be lost [20]. For example, bottom-up techniques may not be able to characterize the coordination in PTM motifs, thus limiting their ability to quantify the stoichiometry among different intact proteoforms. On the other hand, top-down mass spectrometry has been introduced as a comprehensive approach to directly measure and quantify intact proteoforms with PTMs [21–25].

As top-down proteomics methods analyze the intact protein directly, the method is ideal for observing changes in proteoform expression with variable PTM motifs. However, a primary challenge in top-down proteomics is the characterization and quantification of proteoforms in complex systems with high dynamic range [26]. This is particularly challenging for mitochondria; these organelles are characterized by having many protein complexes with low abundance from subpopulations found in the mitochondrial membrane [27–30]. Even still, the Ge group has successfully studied heart tissue and cardiac aging using top-down proteomics and identified proteins related to mitochondrial function [31]. Top-down proteomics has also been used to analyze intact proteins from enriched mitochondria from cultured cells [29, 32, 33].

In this study, we successfully isolated intact mitochondrial proteins from the mouse heart using a trypsin digestion protocol and applied a label-free quantitative top-down proteomics platform to investigate age-related proteoform expression changes. This platform features ultrahigh-pressure liquid chromatography (UPLC)-MS and label-free quantitation, enabling sensitive and high-throughput characterization of proteins. We have applied this platform for comprehensive quantitation of proteoforms expressed in mitochondria collected from young (4 months) and old (24–25 months) mice. In total, we characterized 823 proteoforms from 134 proteins. 20 proteoforms were differentially expressed between the young and old hearts. Among them, 13 proteoforms were decreased in abundance and 7 proteoforms were increased in abundance in the old mice This integrated platform is ideal for characterizing and quantifying intact proteoforms in complex biological samples including difficult-to-study systems such as the mitochondrial proteome.

## METHODS

### Chemicals and Materials.

Pierce^™^ BCA protein assay kit, Halt^™^ Protease Inhibitor Cocktail 100X, Halt^™^ Phosphatase Inhibitor Cocktail, tris(2-carboxyethyl) phosphine hydrochloride (TCEP), and n-dodecyl β-D-maltoside (DDM) were obtained from Thermo Fisher (Waltham, MA). Fetal bovine serum was obtained from Fisher Bioreagents. LC-MS grade 2-propanol, acetonitrile, water, trifluoroacetic acid, sucrose, HEPES, EDTA, trypsin (TG522), trypsin inhibitor (T9201), and other chemicals were purchased from Sigma-Aldrich (St. Louis, MO) unless noted otherwise.

### Animals.

Male C57Bl6 mice were group-housed and maintained on a 14-h light/ dark cycle. The mice in the young group were 4 months old and the mice in the old group were 24–25 months old. Three mice per age group were used for the study. For sample collection, mice were anesthetized with isoflurane and the hearts were excised for mitochondria isolation. All animal procedures were performed based on the Guide for the Care and Use of Laboratory Animals and were approved by the Oklahoma Medical Research Foundation Animal Care and Use Committee.

### Cardiac mitochondria isolation.

Mitochondria were isolated using a trypsin digestion protocol [34]. Briefly, fresh ventricular tissue from a single heart was washed and minced in ice-cold isolation buffer (0.3 M sucrose, 10 mM sodium HEPES, pH 7.2, and 0.2 mM EDTA). The tissue was subjected to trypsin digestion (1.25 mg) for 10 min at 4°C and then diluted with an isolation medium (pH 7.4) containing 0.1% BSA and 2.5 mg of trypsin inhibitor. Tissue was homogenized using 4 strokes of a glass/Teflon potter and drill, set to 300 rpm, in 5x tissue volume of complete homogenization buffer (0.3 M sucrose, 10 mM sodium HEPES, pH 7.2, and 0.2 mM EDTA, 0.1% BSA). The homogenate was centrifuged for 10 min at 800 *g* (4°C). The supernatant solution was decanted and centrifuged for 10 min at 8,000 *g* (4°C). The supernatant was discarded, and the pellet was twice resuspended in isolation medium, and centrifuged for 10 min at 8,000 *g* each time (4°C). The final washed pellet was re-suspended in an isolation buffer with Halt^™^ Protease Inhibitor (1X), Halt^™^ phosphatase inhibitor (1X), trichostatin (0.1μM), and nicotinamide (1μM). Protein concentration was determined by bicinchoninic acid assay (BCA). Isolated mitochondria were snap-frozen in liquid nitrogen and stored at − 80°C until use.

### Lysis of the mitochondria.

The isolated mitochondria from three young and three old mice were resuspended in lysis buffer (20 mM HEPES, 150 mM KCl, 10 mM MgCl_2_, 1 μM PMSF, 0.05% DDM, pH 7.5) and lysed using sonication. The mitochondrial lysate was then centrifuged at 10,000 rpm for 30 minutes at 4°C, and the supernatant was collected.

### Denaturation, reduction, and alkylation of intact mitochondrial protein.

To the mitochondrial lysate, an equal volume of 8 M urea was added so that the final urea concentration was 3 M. After denaturation, the proteins in the lysate were reduced by adding 0.5 M TCEP and incubated at room temperature for 15 minutes. The proteins were alkylated by adding 375 mM iodoacetamide (IAA) (final concentration ~ 20 mM). This solution was incubated at room temperature in the dark for 30 minutes. The denatured, reduced, and alkylated mitochondrial lysate was subjected to ultracentrifugation at 40,000 rpm for 1 hour, and the supernatant was collected. The protein concentration was determined using the Pierce^™^ BCA protein assay kit. Lysate was stored at −80°C until analysis.

### Top-down LC-MS/MS Analysis.

To optimize MS conditions, we performed 9 test runs. Then we performed 12 quantitative runs arising from 3 biological replicates in two conditions (young *vs* old), with technical duplicates for each sample. A modified Thermo Scientific (Waltham, MA) Accela LC system was used for all the runs [6, 7]. 5 μg of the protein from each sample was loaded onto a home-packed C5 trapping column (150 μm i.d., 5 cm length, Jupiter particles, 5 μm diameter, 300 Å pore size) and then separated using a C4 RPLC Capillary column (100 μm i.d., 60 cm length, 3.4 μm diameter, 300 Å pore size) with a flow rate of 200 nL/ min. The composition of mobile phase A (MPA) was 0.01% TFA, 0.585% acetic acid, 2.5% 2-propanol, and 5% acetonitrile in water, and that of mobile phase B (MPB) was 0.01% TFA, 0.585% acetic acid, 45% 2-propanol, and 45% acetonitrile in water. Orbitrap Exploris 240 mass spectrometer (Thermo Fisher Scientific, Bremen, Germany) with a customized nano-ESI interface was used to analyze the LC eluent. A 100-min gradient from 10–70% of MPB was applied. MS parameters were set as follows: inlet capillary temperature was 275°C, spray voltage was 3.0 kV, and resolution for MS1 and MS2 was set to 120000 and 60000 respectively, AGC target was 1 × 10^6^ with 2 micro scans for MS1 and 4 micro scans for MS2. Maximum injection time was 500 ms for MS1 and 300 ms for MS2 scans. The isolation window was set as 2 m/z, the dynamic exclusion window was 90 s, and the top six most abundant precursor ion peaks (charge 4–50) from each MS1 scan were selected for MS2 fragmentation with 35% as normalized higher energy collision dissociation (HCD) energy.

### Data Analysis.

The results of these 21 runs (9 test runs and 12 quantitative runs) were used to create a proteoform identification library (Supplementary Table 1). TopPIC Suite (version 1.4.10) [35] was used to identify the proteoforms and searched against the annotated *Mus Musculus* database (Uniprot 2023-03-24, 17141 species). For TopPIC decoy database searching was used with a maximum number of mass shifts set as 2, the FDR cutoff was set as 0.01 for both spectrum and proteoform levels, and the alkylation on cysteine residues was used as a fixed modification. All other parameters are set as default. The identification library was generated by combining all the identified proteoforms from all datasets. ProSight Lite was used for manual interpretation and spectrum presentation [36].

The collected top-down datasets were deconvoluted and quantified using Biopharma Finder (Thermo Fisher Scientific). An in-house Python software was written for label-free quantitation. This software utilizes an accurate mass and time (AMT) approach to merge mass features between runs [24, 37]. The mass features from different runs were combined and filtered with ± 10 ppm mass shift and ± 5 min retention time shift. The proteoform library was merged with the quantified mass features. The reported results are confirmed with manual evaluation.

## RESULTS

### Identification of intact mitochondrial proteoforms in young and old mice.

**Figure 1:**
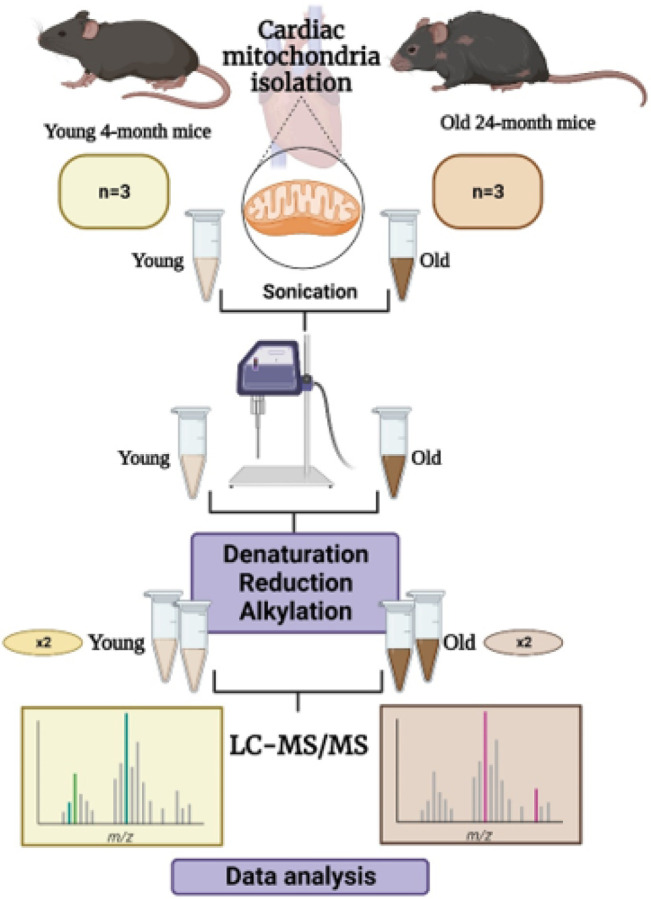
Schematic for intact mitochondrial proteome profiling using quantitative top-down proteomics.

The experimental design for the top-down proteomic analysis of intact mitochondrial proteins is shown in Fig. 1. Intact protein lysate was extracted from purified mitochondria of 3 young (4 months) and 3 old (24–25 months) mice [34]. 5 μg of the extracted mitochondrial proteins were analyzed using LC-MS/MS in technical duplicate. TopPIC Suite was used for protein identification.

**Figure 2:**
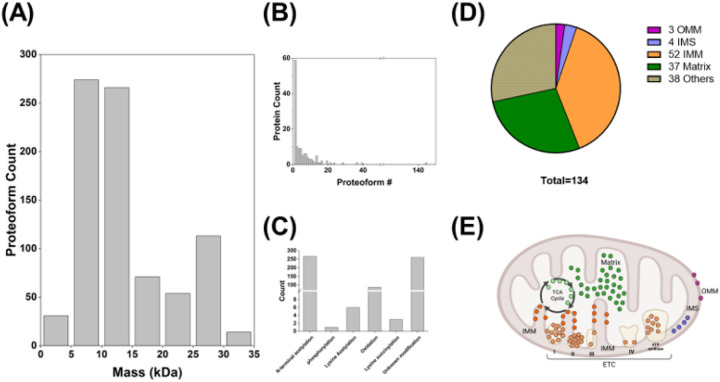
Identification of intact proteoforms from enriched cardiac mitochondria. (A) Histogram demonstrating the mass distribution of uniquely identified proteoforms; (B) Bar graph demonstrating the number of unique proteoforms identified per protein; (C) Bar graph depicting the number of PTMs identified; (D) Pie chart demonstrating the location of the identified proteins in the mitochondria and intercellular space; (E) Visualization of the identified protein in the mitochondria.

In total, we identified 823 unique proteoforms of 134 unique mitochondrial proteins (Supplementary Table 1). These proteoforms ranged in size from ~ 3 kDa to nearly 35 kDa (Fig. 2A). Approximately half of the proteins were expressed as only one proteoform; however, many proteins were expressed as multiple proteoforms and, impressively, one protein had more than 140 characterized proteoforms (Fig. 2B). These proteoforms demonstrated a diverse set of PTMs including acetylation (*N*-terminal acetylation and lysine acetylation), succinylation, acetylation, oxidation, and phosphorylation (Fig. 2C). Many proteoforms (260) carry unknown modifications, either from a combination of multiple PTMs that cannot be distinguished by the MS/MS, or from novel PTMs that have not been well studied.

Further, we used Mito Carta 3.0 and UniProt to determine if the proteins are localized in the mitochondria and categorize the sub-organelle localization of the identified mitochondrial proteins, Fig. 2D&E [5]. Most identified proteins are localized in the mitochondria (96 out of 134 proteins; 72%) and a majority of them (52 proteins out of 96 mitochondrial proteins) are localized in the inner mitochondrial membrane (IMM). Of these, 28 of the identified proteins are components of the oxidative phosphorylation (OXPHOS) complexes. These OXPHOS proteins include subunits from all five complexes (Complex I-V). The second largest sub-organelle class of identified proteins is proteins located in the mitochondrial matrix. This class includes a small group of matrix proteins involved in the TCA cycle. In addition, 4 proteins located in the intermembrane space (IMS) and 3 proteins located in the outer mitochondrial membrane (OMM) were also identified.

### Quantitative comparison of intact mitochondrial proteoforms in young and old hearts.

Biopharma Finder (ThermoFisher Scientific) was used to deconvolute and quantify the identified mass features. Student’s *t*-tests were used to determine the statistical significance of the fold change of each proteoform between the young and old hearts. To be considered statistically significant the p-value should be less than 0.05 (95% confidence). Fold change was calculated using the ratio of average intensities of the mass features in both conditions.

**Figure 3:**
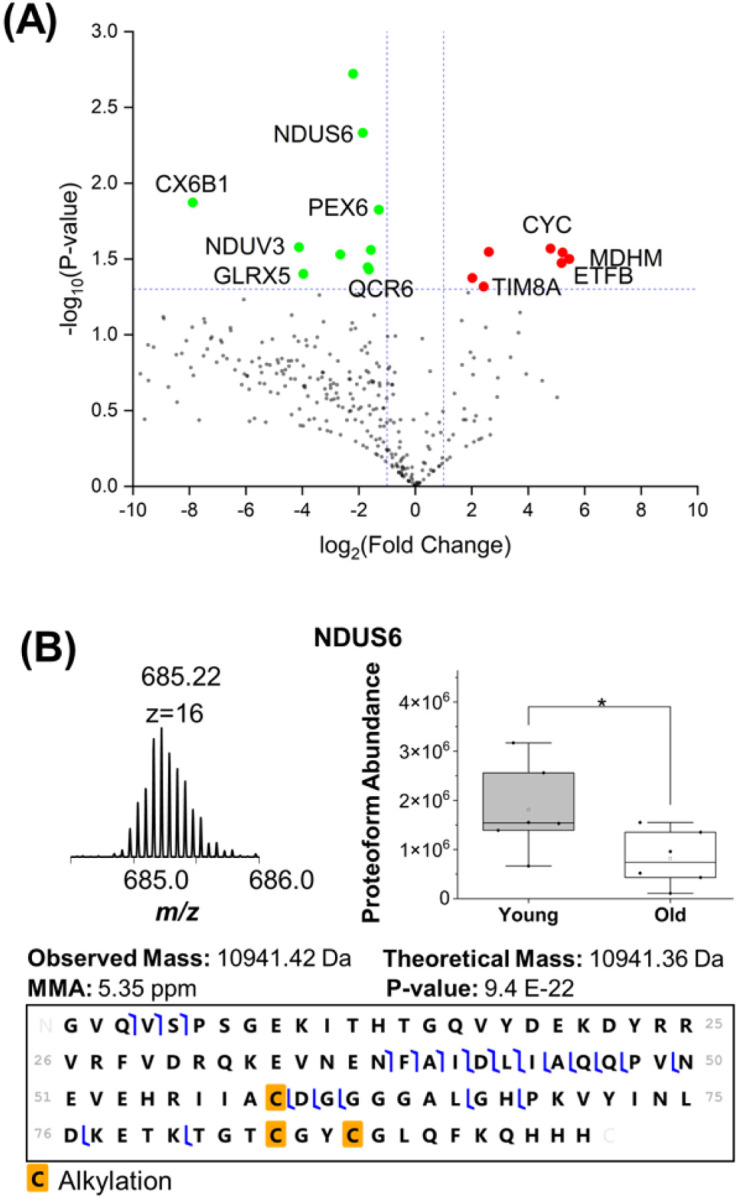
Examination of the change in intact mitochondrial proteoform abundance as a function of age. (A) Volcano plot demonstrating the differential expression of identified proteoforms. The vertical dotted lines represent a log2 fold change cutoff of 1, and the horizontal dotted line represents a – log10 p-value cutoff of 1.3. The proteoforms that were increased in the old mouse samples are shown in red and the decreased proteoforms are shown in green. (B) expression of NADH dehydrogenase [ubiquinone] iron-sulfur protein 6 (NDUS6; P52503) was found to be significantly reduced in old mitochondria samples.

A volcano plot was created to visualize the differential expression of proteoforms between young and old hearts (Fig. 3A). Overall, 20 proteoforms were differentially expressed between the young and old hearts. Thirteen (13) proteoforms were decreased in abundance and 7 proteoforms were increased in abundance in the old mice (Supplementary Table 2). For example, the expression of NADH dehydrogenase [ubiquinone] iron-sulfur protein 6 (NDUS6; P52503), an accessory subunit of Complex I, was significantly reduced in old hearts compared to young hearts (Fig. 3B). Other mitochondrial proteins that had identified proteoforms with decreased abundance in cardiac mitochondria from the old mouse group included cytochrome c oxidase subunit 6B1 (CX6B1, P56391), NADH dehydrogenase [ubiquinone] flavoprotein 3 (NDUV3, Q8BK30), glutaredoxin-related protein 5 (GLRX5, Q80Y14), cytochrome b-c1 complex subunit 6 (QCR6, P99028). Other proteins that increased in abundance in the old mouse samples included mitochondrial import inner membrane translocase subunit Tim8 A (Tim8A, Q9WVA2), cytochrome c, somatic (CYC, P62897), electron transfer flavoprotein subunit beta (ETFB, Q9DCW4), and malate dehydrogenase (MDHM, P08249)

**Figure 4:**
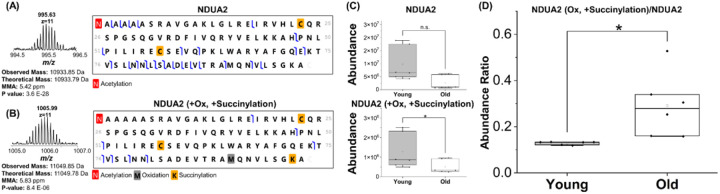
Quantitative analysis of the NADH dehydrogenase 1 alpha subcomplex subunit 2 (NDUA2, Q9CQ75) protein. (A-B) Isotopic distribution and fragment map of the identified proteoforms. (C) Box and whisker plots demonstrating the abundance of each proteoform in young and old mouse samples. (C) The ratio between different proteoforms in young and old mouse samples.

In addition to changes in protein abundance, we have investigated PTM-specific changes with aging. For instance, we identified 3 different proteoforms of NADH dehydrogenase ubiquinone 1 alpha subcomplex subunit 2 (Q9CQ75). The most abundant proteoform was acetylated at the *N*-terminal (Fig. 4A). Another proteoform was expressed at lower abundance and was acetylated at the N-terminal and oxidized at Met-90 (*data not shown*). This proteoform was present at very low intensity and was not quantifiable in this dataset. Oxidation of intact proteoforms can be endogenous or artificial, however, in this case, the abundance of the oxidized proteoform is negligible. The remaining identified proteoform was acetylated at the *N*-terminal (Fig. 4B), oxidized at Met-90, and succinylated at Lys-97. No intact proteoforms were detected that were only succinylated at Lys-97 with no oxidation at Met-90. Both the *N*-terminally acetylated proteoform and the oxidized and succinylated proteoform showed a decreasing abundance trend in the old mice, although this change in expression was only significant for the succinylated and oxidized proteoform, Fig. 4C. Interestingly, the ratio of the oxidized and succinylated proteoform compared with the *N*-terminally acetylated proteoform was shown to significantly increase in the old mice, Fig. 4D. This protein has been previously reported to be succinylated at Lys-64 [38]; however, our results show that lysine residue 97 can also be succinylated.

**Figure 5:**
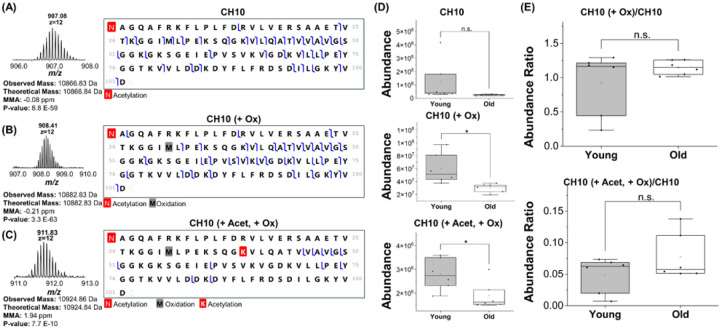
Quantitative analysis of the 10 kDa Heat shock protein (CH10, Q64433) protein. (A-B) Isotopic distribution and fragment map of the identified proteoforms. (C) Box and whisker plots demonstrating the abundance of each proteoform in young and old mouse samples. (D) The ratio between different proteoforms in young and old mouse samples.

Three proteoforms of the 10 kDa Heat Shock protein, mitochondrial (CH10, Q64433) were identified. One proteoform was acetylated at the *N*-terminal (Fig. 5A), the second proteoform maintained *N*-terminal acetylation and was also oxidized at Met-31 (Fig. 5B), and the third proteoform maintained the previously mentioned modifications and was also acetylated at Lys-39 (Fig. 5C). This lysine has previously been reported to be acetylated in the Uniprot database [39]. Overall, the expression of all three proteoforms for this protein trended down in the old mice, although only the second and the third proteoforms were significantly decreased (Fig. 5D). Interestingly, when the ratio of these two proteoforms normalized to the *N*-terminally acetylated proteoform was examined, we found that the ratio of these two proteoforms did not decrease (Fig. 5D).

These two examples show that changes in stoichiometry in different PTM-modified intact proteoforms may not be reflected by the expression of a single proteoform or a single peptide. On the other hand, these changes, even for proteoforms with combinatorial PTMs, can be readily observed for intact proteoforms analyzed using top-down proteomics. This, however, may not be possible using bottom-up proteomics data.

## Discussion

Aging is a major risk factor for the development of cardiac disease. The heart is a highly metabolic organ and its proper functioning is highly dependent on healthy mitochondria to supply sufficient energy [40]. The study of age-related mitochondrial changes has been an important research field for many years; however, the dynamic range in protein abundance of mitochondrial proteins has made it difficult to study intact proteoforms with PTMs that may be related to metabolic function. Bottom-up proteomics techniques are sensitive and robust and can be used for the high-throughput, quantitative study of complex biological systems including mitochondrial proteins [41, 42]. However, bottom-up proteomics methods require protein digestion which can obscure important information regarding the intact proteoform including PTM motifs and other modifications [22, 43].

Top-down proteomics analyzes the intact proteoforms directly so information regarding protein modifications is maintained. However, previous applications of top-down proteomics to complex biological systems have been challenging due to the relatively low sensitivity of the methods and the high dynamic range of the samples. Improvements to liquid chromatography [21, 44–46] and mass spectrometry [47] methods concerning separation efficiency and sensitivity have allowed the application of top-down proteomics to important biological systems such as age-related systems. These new applications can give us new insight into the pathophysiological mechanisms of age-related cardiovascular disease [48, 49]. Using our developed label-free quantitative top-down proteomics UPLC-MS/MS platform [50], we have studied, for the first time, age-related differential expression of mitochondrial proteoforms extracted from enriched cardiac mitochondria in mice.

In this survey study, we identified a total of 134 proteins in the samples and 96 of them (72%) were mitochondrial proteins (Fig. 2D). This result validated the successful enrichment of mitochondrial proteins by our mitochondrial isolation method and demonstrated the compatibility of the enrichment method with top-down proteomics analysis. Moreover, we identified mitochondrial proteins located in all sub-mitochondrial compartments (OMM, IMS, IMM, matrix), supporting that intact mitochondria were isolated. Proteins located in the IMM comprise over 50% of the mitochondrial proteins identified. Among the 52 IMM proteins detected, 28 of them are OXPHOS proteins. This high percentage is consistent with the high abundances of OXPHOS proteins which represent two-thirds of the total proteins of the IMM. On the other hand, we detected a low number of proteins located in the IMS and OMM. The lower number of proteins detected in these sub-mitochondrial compartments is consistent with the lower numbers of proteins present in these compartments. Specifically, OMM proteins account for approximately 10% of all mitochondrial proteins, and IMS proteins represent about 5% of all mitochondrial proteins. [5]

Using label-free quantitative top-down proteomics, we detected age-related changes in the abundance of intact proteoforms in heart mitochondria (Fig. 3A). Four proteins (NDUS6, NDUV3, CX6B1, and OCR6) that exhibited reduced abundance with aging are subunits of the ETC complex, and our result is consistent with previous findings on aging-associated decline in energy metabolism and reduced expression of genes coding for oxidative phosphorylation mitochondrial proteins [51, 52]. Another protein with reduced expression with aging is glutaredoxin 5 (GLRX5), a mitochondrial glutaredoxin involved in iron/sulfur protein assembly [53]. GLRX5 has been shown to protect against oxidative stress in yeast and osteoblasts [54, 55]. The reduced expression of GLRX5 in old hearts may in part contribute to the increase in mitochondrial oxidative stress in the aging heart [3].

For proteins that were detected with increased abundance with aging, TIM8A is a small Tim chaperone protein involved in the assembly of Complex IV [56]. A previous study has shown that TIM8A expression decreases when a GSK inhibitor induces cardioprotection [57]. Whether increased TIM8A contributes to cardiac dysfunction in the aging heart remains to be investigated. We also detected an age-related increase in the level of ETFB, a flavoprotein involved in mitochondrial metabolism. ETFB is associated with anthracycline-mediated mitochondrial dysfunction in cardiotoxicity in cancer patients but its role in mitochondrial dysfunction in cardiac aging is unclear [58].

Cardiac proteins are continuously synthesized and degraded to ensure protein homeostasis. In addition to dynamic changes in protein expression, PTMs of proteins offer another regulatory mechanism to fine-tune protein function and in turn, modulate different cellular processes. PTMs of proteins can regulate protein folding, half-life of proteins, and protein-protein interactions [59]. Multiple post-translational modifying enzymes have been identified in the mitochondria, and several PTMs have been described in mitochondria [60].

In this study, we detected three main PTMs, oxidation, acetylation, and succinylation, in cardiac mitochondrial proteins. Increased protein oxidation due to high ROS production is an age hallmark of cardiac aging and contributes to the dysregulation of mitochondrial dynamics and protein quality control [61]. Lysine acetylation and succinylation are two forms of protein acylation modifications. Mitochondrial protein acetylation and succinylation can be removed by NAD^+^-dependent deacetylases sirtuins SIRT3 and SIRT5 [62, 63]. NAD^+^ depletion, reduced SIRT3 and SIRT5 activity and increased lysine acylation have been implicated as pathogenic mechanisms in CVDs [64, 65]. Our top-down proteomic analysis quantifies changes in PTMs on intact mitochondrial proteoforms and allows us to study combinatorial changes in these PTMs. Although we detected no difference (or even a decreasing trend) in the absolute levels of succinylated NDUA2 in young and old hearts, we observed an increased ratio of succinylated/un-succinylated NDUA2 proteoforms, suggesting increased succinylation of NDUA2 with aging (Fig. 4C and 4D). This result highlights the importance of normalization to the level of un-modified proteoform when investigating the change in a specific PTM.

Despite the enrichment of mitochondrial proteins, we still detected proteins located in other subcellular compartments, suggesting the presence of non-mitochondrial contaminants such as peroxisomes, ER, and Golgi. [66]. LC-MS/MS is a sensitive technique that can detect small amounts of contaminating proteins. Physical interaction between mitochondria and these organelles makes the purification process challenging; complementing differential centrifugation with another separation technique could enhance the purity of the mitochondrial fraction [67, 68].

In this study, intact mitochondrial proteoforms were extracted using an aqueous buffer omitting any detergent to solubilize insoluble hydrophobic proteins such as membrane proteins. As the structure of mitochondria involves a complex membrane system with many membrane-bound proteins, a biological limitation of this technique is the inability to characterize insoluble membrane proteins. Previous studies have analyzed intact mitochondrial membrane proteins from cultured cells [33]. Recently, there have been reports of MS-compatible detergents for top-down proteomics analysis of insoluble proteins [69, 70]. Future application of these MS-compatible detergents could allow top-down proteomics analysis of intact proteoforms from mitochondria enriched from tissue.

Further improvements regarding intact protein separation efficiency, quantitation, and MS sensitivity could improve proteoform characterization and quantitation for this method. For example, other separation techniques such as capillary electrophoresis that have higher sensitivity and separation efficiency compared with LC methods have been implemented for top-down proteomics [71–74]. Additionally, multidimensional separation of intact proteins has been shown to improve separation efficiency and allow for the characterization of more intact proteoforms in complex samples [21, 75–81]. However, label-free quantitation techniques as used here are challenging to implement with multidimensional separation methods. The application of isobaric chemical tag labeling allows protein quantitation in multidimensional separations [22]. Fortunately, recent reports have demonstrated the development of isobaric chemical tag labeling methods for intact proteins [30, 82–87], and coupling these techniques could greatly improve quantitative top-down proteomics methods. Finally, high-field asymmetric waveform ion mobility spectrometry (FAIMS) methods have been applied for intact protein MS analysis to reduce noise and improve the signal-to-noise ratio [73, 74].

## CONCLUSION

Altogether, we have shown the potential of quantitative top-down proteomics techniques to identify and quantify, for the first time, age-related changes in mitochondrial proteoforms directly from enriched mitochondria from the heart. Analysis of proteoforms in mitochondria samples has traditionally been difficult because of low proteoform abundance and high dynamic range; however, with our enrichment strategy of subcellular fractionation coupled with our ultrahigh-pressure separation coupled with sensitive MS detection, we identified hundreds of intact proteoforms from more than 100 proteins from different sub-mitochondrial compartments. In addition to identifying age-related changes in protein abundance, our top-down proteomic analysis provides a bird’s eye view of combinatorial PTMs in intact mitochondrial proteoforms. Overall, this survey study demonstrates the capabilities of intact mitochondrial proteoform characterization and quantification by enriching mitochondria from cardiac tissue followed by highly sensitive UPLC-MS/MS analysis.
